# Inappropriate dispensing of antibiotics in urban and rural pharmacies in Dhaka: A cross-sectional observation

**DOI:** 10.1016/j.aprim.2025.103342

**Published:** 2025-12-08

**Authors:** Md. Monir Hossain Shimul, Farjana Ajmary Haque, Salamat Khandker

**Affiliations:** Department of Public Health, Daffodil International University, Dhaka, Bangladesh

The rise of antimicrobial resistance (AMR) has become a public health emergency globally, with irrational use of antibiotics being a primary driver particularly in low- and middle-income countries (LMICs) like Bangladesh, where over-the-counter antibiotic sales remain alarmingly high despite legal prohibitions.^1^ This study aimed to assess the pattern of antibiotic sales and identify the economic and behavioral factors contributing to inappropriate antibiotic dispensing in both urban and rural private pharmacies of Dhaka.

From September to December 2024, an observational cross-sectional study was conducted in 246 randomly selected pharmacies across Dhaka. Data were collected on licensing status, staff qualifications, sales behaviors, types of antibiotics sold, and reasons behind irrational dispensing. Pharmacy owners and dispensers were informed in advance, but observations were carried out for all drug sales to reduce observer bias. A structured checklist and observational form were used to record variables across pharmacy type, sales pattern, and the presence of a pharmacist. Data were analyzed using SPSS v26.

Among the 246 pharmacies observed, 93.48% held a valid license, but only 7.56% had a registered pharmacist present. A substantial proportion (73.59%) was located in urban areas. Despite the legal requirement, nearly 45% of antibiotics were dispensed without prescription (self-medication), and another 19.17% were dispensed based on informal consultation with dispensers. Only 36.24% were sold with a registered physician's prescription. Self-medication was marginally more common in rural pharmacies (47.69%) than in urban ones (43.65%), a trend consistent with findings from other LMICs.^2^

The most frequently dispensed antibiotic class was penicillin (37.29%), followed by quinolones (18.38%) and macrolides (17.83%). In rural areas, penicillin sales were higher (41.54%) compared to urban pharmacies (35.76%), a pattern aligned with studies in similar settings.^3^

Inappropriate dispensing was driven by a complex interplay of socioeconomic and systemic factors. The leading reason was fear of losing customers, reported by 99.54% of rural and 74.59% of urban pharmacies. Pressure from patients (rural 77.65%, urban 65.32%), lack of adequate healthcare providers, particularly in rural areas (65.49%), and high profitability of antibiotics were other notable drivers (Table 1). Alarmingly, only 25.34% of rural and 21.02% of urban pharmacies cited insufficient knowledge as a cause, reflecting low awareness of the long-term impact of AMR.^4^

**Table 1:** Summary of key findings on pharmacy characteristics, sales practices, and drivers of inappropriate antibiotic dispensing (*N* = 246).

Variable	Rural (*n* = 65)	Urban (*n* = 181)	Total (*N* = 246)
Licensed Pharmacies	58 (89.2%)	172 (95.0%)	230 (93.5%)
Pharmacist Present	3 (4.6%)	15 (8.3%)	18 (7.3%)
Antibiotic Sales by Self-Medication	31 (47.7%)	79 (43.6%)	110 (44.6%)
Antibiotic Sales with Prescription	22 (33.9%)	67 (37.0%)	89 (36.2%)
Most Common Antibiotic: Penicillin	27 (41.5%)	65 (35.8%)	92 (37.4%)
Fear of Losing Customer	65 (100.0%)	135 (74.6%)	200 (81.3%)
Pressure from Patients	50 (76.9%)	118 (65.2%)	168 (68.3%)
Insufficient Doctors in Health System	43 (66.2%)	56 (30.9%)	99 (40.2%)
High Profitability of Antibiotics	19 (29.2%)	82 (45.3%)	101 (41.1%)
Patients’ Reluctance to Visit Doctors	23 (35.4%)	39 (21.5%)	62 (25.2%)
Insufficient Knowledge among Dispensers	16 (24.6%)	38 (21.0%)	54 (22.0%)
Polypharmacy Practices	8 (12.3%)	11 (6.1%)	19 (7.7%)
Inappropriate Prescriptions by Providers	5 (7.7%)	4 (2.2%)	9 (3.7%)

This study adds to the growing body of literature revealing the disconnect between regulatory frameworks and real-world pharmacy practices in LMICs.^5^ While Bangladesh has formal laws prohibiting over-the-counter antibiotic sales, enforcement remains weak, and economic incentives overshadow public health considerations. Community-level awareness, training of drug sellers and stricter implementation of good pharmacy practice (GPP) guidelines are urgently needed.^6^

In conclusion, inappropriate antibiotic dispensing is widespread in Dhaka's private pharmacies, driven by economic pressure, insufficient healthcare access, and regulatory failures. Tailored interventions such as mandatory pharmacist presence, patient education, and antibiotic stewardship programs are essential to curb this public health threat.

## Author contributions

1. Md. Monir Hossain Shimul: Conceptualization, formal analysis, investigation, methodology, visualization, writing – original draft, and writing – review & editing.
2. Farjana Ajmary Haque: Conceptualization, data curation, formal analysis, methodology, writing – original draft.
3. Salamat Khandker: Methodology, supervision, writing – review and editing.

## Ethical considerations

The study was conducted in compliance with ethical guidelines, with approval obtained from the Research Ethics Committee of the Faculty of Health and Life Sciences, Daffodil International University (Approval Reference: FHLSREC/DIU/2024/SMIG-156). Written consent has been obtained from all participants.

## Transparency declaration

The corresponding author, in the name of the rest of the signatories, declares that the data and information contained in the study are precise, transparent and honest; that no relevant information has been omitted; and that all the discrepancies among authors have been adequately resolved and described.

## Repository

Not applicable.

## Funding

This research did not receive any specific grant from funding agencies in the public, commercial, or not-for-profit sectors.

## Conflict of interest

The authors and corresponding authors declare no competing interests.
